# Traditional Chinese medicine on treating active rheumatoid arthritis: A protocol for systematic review and meta-analysis

**DOI:** 10.1097/MD.0000000000020642

**Published:** 2020-06-12

**Authors:** Lingyue Zhang, Zuoyuan Cao, Yeying Yang, Xinyi Tan, Jianchun Mao, Li Su

**Affiliations:** Longhua Hospital, Shanghai University of Traditional Chinese Medicine, Shanghai, China.

**Keywords:** active rheumatoid arthritis, protocol, systematic review, traditional Chinese medicine

## Abstract

**Background:**

Rheumatoid arthritis (RA) is a chronic systemic autoimmune disease associated with progressive joint damage and disability. There is a lack of effective methods in the treatment of RA currently. Many clinical trials have proved that traditional Chinese medicine (TCM) has obvious advantages in the treatment of RA. In this systematic review, we intend to evaluate the efficacy and safety of TCM for active RA.

**Methods:**

We will search PubMed, the Cochrane Library, Embase, Web of Science, the Chinese National Knowledge Infrastructure Database, Wanfang Data, and Chinese Science and Technology Periodical Database. Simultaneously we will retrieval relevant meeting minutes, eligible research reference lists, symposium abstracts, and grey literatures. Included criteria are randomized controlled trials (RCTs) about TCM for active RA to assess its efficacy and safety. We will use the Revman 5.3 and Stata 13.0 software for data synthesis, sensitivity analysis, meta regression, subgroup analysis, and risk of bias assessment. The Grading of Recommendations Assessment, Development, and Evaluation standard will be used to evaluate the quality of evidence.

**Results:**

This systematic review will provide a synthesis of TCM for patients with active RA from various evaluation aspects including tender joint count, swollen joint count, RF, CRP, ESR, DAS28, TCM syndrome evaluation criteria, and adverse events.

**Conclusion:**

The systematic review will provide evidence to assess the efficacy and safety of TCM in the treatment of patients with active RA.

**PROSPERO registration number:**

PROSPERO CRD42019146726

## 1 Introduction

Rheumatoid arthritis (RA) is a chronic autoimmune inflammatory disease of joints characterised by pain, stiffness, inflammation, and disability.^[1]^ The global prevalence of RA is estimated at 0.5% to 1%, second only to osteoarthritis and gout as major causes of disability.^[2]^ Currently, antirheumatic drugs include glucocorticosteroids, nonsteroidal anti-inflammatory drugs (NSAIDs), disease-modifying antirheumatic drugs (DMARDs), and biological agents (e.g., anti-TNF-therapy). As the main treatment for RA, DMARDs is used to relieve joint pain and swelling, and to control disease activity.^[3]^ Although DMARD therapy is essential to obtain disease control, long-term use comes with several disadvantages. Apart from obvious defects such as drug toxicity and side effects, medication use by itself may be considered as burdensome and unhealthy by patients.^[4]^ Biologic agents are typically used in patients with severe unresponsive disease to the classical DMARDs. Although effective, they are significantly expensive, imposing a heavy economic cost on the patients and on society in China.^[5]^

RA belongs to the category of “Bi Zheng” (Bi syndrome) in traditional Chinese medicine (TCM). In the past thousands of years, TCM has provided many useful and inexpensive methods for RA therapy.^[6]^ Modern researchers have done a lot of experimental studies on the mechanisms of TCM and made great progress. Various herbals and herbal formulas or extracts, such as Guizhi-Shaoyao-Zhimu decoction, Wu-Tou Tang, and extracts of the herb Tripterygium wilfordii Hook, F., are proved to be beneficial for alleviating RA progression.^[7–9]^ TCM regulates body function as a whole through multifaceted and multi target mechanism, and has obvious advantages in the treatment of RA.

In recent years, TCM for RA therapy has become a research hotspot, and related randomized controlled trials (RCTs) has also increased. The purpose of this systematic review and meta-analysis is to assess the clinical efficacy and safety of TCM in the treatment of active RA and to provide clinicians with a medical basis for inquiry.

## 2 Methods

### 2.1 Inclusion criteria for study selection

#### 2.1.1 Types of studies

All RCTs that evaluate the efficacy and safety of TCM in the treatment of active RA will be incorporated into our study. No restrictions were imposed on study dates or publication language, type, and status.

#### 2.1.2 Types of participants

Patients were selected when fulfilling the inclusion criteria of RA diagnosis (1987 or 2010 ACR/EULAR criteria). Patients had to have obvious swollen and tender joints and at least two of the following: morning stiffness lasting ≥60 min; serum C reactive protein (CRP) concentration and erythrocyte sedimentation rate∗∗∗∗ (ESR) increased significantly (or DAS28>2.6). There were no restrictions for sex, age, race or nationality.

#### 2.1.3 Types of interventions

The drug composition, the dose-specific Chinese medicine preparation or combined with conventional treatment of Western Medicine, is used as experimental interventions. Both prescription and Chinese patent medicines will be included. Other TCM treatments such as intravenous medication, acupuncture, and massage will be excluded. As for the control interventions, those who accepted western medicine alone can be used as a control intervention or those who did not get any treatment as a blank control would be adopted.

### 2.2 Types of outcome measures

#### 2.2.1.1 Major outcomes

- Tender joint count(TJC), Swollen joint count(SJC)
- Changes in rheumatoid factors (RF), C-reactive protein (CRP) and ESR.

#### 2.2.1.2 Secondary outcomes

- Disease Activity Score 28 (DAS28)
  The DAS28 is an index calculating the number of painful and swollen joints (28 joints, i.e., shoulders, elbows, wrists, metacarpophalangeal and proximal interphalangeal joints, and knees), ESR and the score of global assessment.^[10]^ The formula of DAS 28 is 0.56 × √(28 painful joint count) + 0.28 × √(28 swollen joint count) + 0.70 × (ln ESR) + 0.014 × GH. ESR refers to ESR. GH is the patient's general health visual analog scale (0–10 mm).^[11–13]^
- Syndrome according to standards for assessing TCM
- Adverse events

### 2.3 Search methods for the identification of studies

#### 2.3.1 Electronic searches

**Figure 1:**
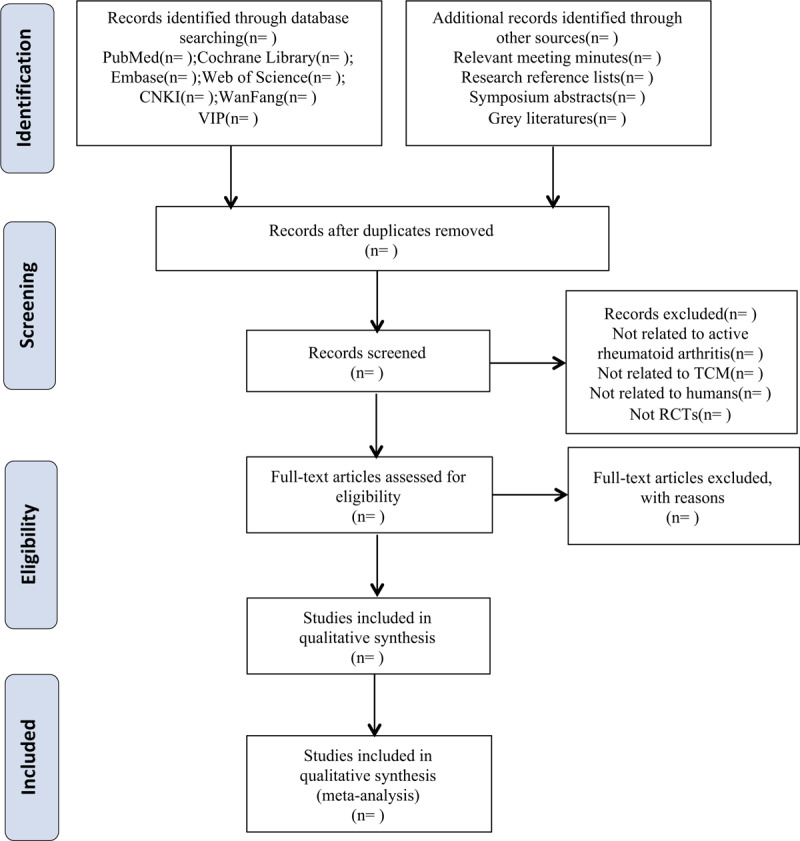
Flow chart of study selection process.

The electronic search database includes PubMed, the Cochrane Library, Embase, Web of Science, the Chinese National Knowledge Infrastructure Database (CNKI), Wanfang Data, and Chinese Science and Technology Periodical Database (VIP). The search terms include “Chinese medicine” or “Traditional Chinese medicine” combined with “active rheumatoid arthritis,” “rheumatoid arthritis” or “RA.” The literatures involved are those delivered from the time when the databases were established to January 2020. The search terms in the Chinese database will be the translations of the above words. A Preferred Reporting Items for Systematic Reviews and Meta-Analyses (PRISMA) flowchart will be created to show the number of articles identified, screened, included, and excluded, reasons for exclusion and to confirm eligible studies. The study selection process will be described in a PRISMA flowchart (http://www.prisma-statement.org) (Fig. 1).

#### 2.3.2 Other resources search

Relevant meeting minutes, eligible research reference lists, symposium abstracts, and grey literature such as degree papers, conference papers will be manually searched for additional resources.

### 2.4 Data acquisition and analysis

#### 2.4.1 Selection of studies

Two researchers will independently screen the titles and abstracts of the selected studies. Also then full texts reading will be completed to select the final literatures that meet the included criteria. The EndnoteX7 software will apply to manage the included references.

#### 2.4.2 Data extraction and management

Two researchers will screen all selected articles and extract the data independently. Excel spreadsheet will be used to extract relevant data, including the first author, year of publication, the observation period, the number of participants, the intervention methods of the treatment group and the control group, outcome measures, study results, and adverse events. If there are disagreements, they will settle through discussion. If necessary, the divergence will be discussed with the third author.

#### 2.4.3 Assessment of risk of bias in included studies

The risk of bias in all included studies will be evaluated by the assessment tool recommended by Cochrane. The assessment will include random sequence generation, allocation concealment, blindness of participants and investigators, blindness of outcome assessment, incomplete outcome data, selective outcome reporting, and other bias. The risk grade will be classified as “low bias,” “high bias,” and “unclear bias.” The evaluation will be independently evaluated by 2 researchers. If there are different opinions, the discussion will be conducted. If there are still differences, a third-party will be consulted.

#### 2.4.4 Measures of treatment effect

For continuous data, the extracted data will be assessed using a standard mean difference (SMD) of 95% confidence interval (95% CI). For dichotomous outcomes, we will choose the effect scale indicator relative risk (RR) with 95% CI to represent.

#### 2.4.5 Missing data

The researchers will contact the first author by email for further information about the studies while there are missing data. If the missing data is still not obtained in the above way, we will analyze the available data. Furthermore, we will also discuss the potential impact of the missing data.

#### 2.4.6 Assessment of heterogeneity

Heterogeneity will be assessed by chi-squared test and *I*^2^ test. If *I*^2^ > 50%, *P* < .1, there is a large heterogeneity between studies. If *I*^2^ ≤ 50%, *P* > .1, there is no statistical heterogeneity between each studies. If there is significant heterogeneity between a set of studies, we will use a random-effects model (REM) for meta-analysis, otherwise, fixed-effect model (FEM) will be used.

#### 2.4.7 Assessment of publication biases

If there are more 10 articles in the meta-analysis, a funnel plot will be established to assess the publication bias. Begg and Egger tests will be used to help assess the symmetry of funnel plot.

#### 2.4.8 Data analysis

Review Manager software version 5.3 and Stata13.0 statistical software will be used for synthesis and analysis of the data. If there is no heterogeneity (*I*^2^ ≤ 50%, *P* > .1), a FEM will be used for analysis. Otherwise (*I*^2^ > 50%, *P* < .1), a REM will be used for meta-analysis. If there is significant clinical heterogeneity, we will use subgroup analysis or sensitivity analysis, or just descriptive analysis.

#### 2.4.9 Subgroup analysis

If the results of the study are heterogeneous, subgroup analysis will be conducted to explore the reasons for the existence of heterogeneity from various aspects, such as the characteristics of the participants, different control interventions, and outcome measures.

#### 2.4.10 Sensitivity analysis

Sensitivity analysis will be used to assess the quality of the included studies based on sample size, statistical method, and missing data.

#### 2.4.11 Grading the quality of evidence

The Grading of Recommendations Assessment, Development, and Evaluation (GRADE) standard will be used to evaluate the quality of evidence. The GRADE system divides the quality of evidence into four levels: high, moderate, low, and very low.^[14]^

#### 2.4.12 Ethics

The ethical approval is not necessary, because we will not use data related to individual patient data.

## 3 Discussion

As a refractory disease, RA results in joint deformity, decrease work ability, productivity and subsequently reduces quality of life of an individual.^[15]^ TCM believes that the etiology and pathogenesis of RA is related to external cause, internal cause, phlegm and static blood. External cause mainly refers to wind, cold, and dampness pathogen. Deficiency of qi, blood, liver, and kidney is the internal cause of RA. TCM can play the role of nourishing the liver and kidney, promoting qi and blood circulation for removing blood stasis. Therefore, TCM has been used to treat RA for more than three thousands of years in China. Modern researches show that the effective active ingredients in Chinese medicine can weak disease activity of RA and protect joints from deformity.^[16,17]^

TCM for RA has broad application prospects. Therefore, we attempt to perform this systematic review and meta-analysis to provide high-quality evidence for the clinical efficacy and safety of TCM in the treatment of RA. There may be some potential shortcomings in this study. For example, languages other than Chinese and English will be restricted, which will lead to some bias. In addition, the variety of age, dosage, and treatment course may result in significant clinical heterogeneity. We also hope that there will be more high-quality RCTs of TCM for RA therapy in the future.

## Author contributions

**Conceptualization:** LingYue Zhang.

**Data curation:** ZuoYuan Cao, XinYi Tan.

**Formal analysis:** LingYue Zhang, ZuoYuan Cao, YeYing Yang.

**Funding acquisition:** LingYue Zhang.

**Methodology:** YeYing Yang, JianChun Mao.

**Project administration:** LingYue Zhang.

**Resources:** LingYue Zhang, ZuoYuan Cao.

**Software:** LingYue Zhang, XinYi Tan.

**Supervision:** Li Su.

**Writing – original draft:** LingYue Zhang.

**Writing – review & editing:** Li Su.
